# Pairing and Exchanging between *Daypyrum villosum* Chromosomes 6V#2 and 6V#4 in the Hybrids of Two Different Wheat Alien Substitution Lines

**DOI:** 10.3390/ijms20236063

**Published:** 2019-12-01

**Authors:** Xiaolan Ma, Zhiying Xu, Jing Wang, Haiqiang Chen, Xingguo Ye, Zhishan Lin

**Affiliations:** 1 Institute of Crop Sciences, Chinese Academy of Agricultural Sciences, Beijing 100081, China; 13264529812@163.com (X.M.); 13414916566@163.com (Z.X.); 13121260899@163.com (J.W.); haiqiang_chen@163.com (H.C.); yexingguo@caas.cn (X.Y.); 2 Agricultural College, Guangdong Ocean University, Zhanjiang 524088, China; 3 National Key Facility of Crop Gene Resources and Genetic Improvement, Chinese Academy of Agricultural Sciences, Beijing 100081, China

**Keywords:** wheat, *Dasypyrum villosum*, alien substitution line, GISH, molecular marker, marker-assisted selection

## Abstract

Normal pairing and exchanging is an important basis to evaluate the genetic relationship between homologous chromosomes in a wheat background. The pairing behavior between 6V#2 and 6V#4, two chromosomes from different *Dasypyrum villosum* accessions, is still not clear. In this study, two wheat alien substitution lines, 6V#2 (6A) and 6V#4 (6D), were crossed to obtain the F_1_ hybrids and F_2_ segregating populations, and the testcross populations were obtained by using the F_1_ as a parent crossed with wheat variety Wan7107. The chromosomal behavior at meiosis in pollen mother cells (PMCs) of the F_1_ hybrids was observed using a genomic in situ hybridization (GISH) technique. Exchange events of two alien chromosomes were investigated in the F_2_ populations using nine polymerase chain reaction (PCR) markers located on the 6V short arm. The results showed that the two alien chromosomes could pair with each other to form ring- or rod-shaped bivalent chromosomes in 79.76% of the total PMCs, and most were pulled to two poles evenly at anaphase I. Investigation of the F_2_ populations showed that the segregation ratios of seven markers were consistent with the theoretical values 3:1 or 1:2:1, and recombinants among markers were detected. A genetic linkage map of nine PCR markers for 6VS was accordingly constructed based on the exchange frequencies and compared with the physical maps of wheat and barley based on homologous sequences of the markers, which showed that conservation of sequence order compared to 6V was 6H and 6B > 6A > 6D. In the testcross populations with 482 plants, seven showed susceptibility to powdery mildew (PM) and lacked amplification of alien chromosomal bands. Six other plants had amplification of specific bands of both the alien chromosomes at multiple sites, which suggested that the alien chromosomes had abnormal separation behavior in about 1.5% of the PMCs in F_1_, which resulted in some gametes containing two alien chromosomes. In addition, three new types of chromosome substitution were developed. This study lays a foundation for alien allelism tests and further assessment of the genetic relationship among 6V#2, 6V#4, and their wheat homoeologous chromosomes.

## 1. Introduction

Wheat (*Triticum aestivum* L. 2*n* = 6*x* = 42, AABBDD) is one of the most widely cultivated crops in the world. Biotic and abiotic stresses often cause different degrees of decline in wheat yield and quality. There are many genes in wheat relatives with desirable traits, which have great potential to improve the resistance of common wheat to various stresses. For example, *Dasypyrum villosum* (2*n* = 2*x* = 14, VV), which originated in the Mediterranean and the Caucasus region [1,2], is a cross-pollinating annual plant that retained many characteristics that ordinary wheat lacks, such as resistances to powdery mildew (PM), stem rust, leaf rust, leaf blight, and scattered smut [1,2]. *D. villosum* can, therefore, be used as a potential resistance source for wheat breeding [3]. The resistance of the tertiary gene pool of wheat is also non-host resistance [4], which is usually wide spectrum and high efficiency. *Pm21*, a resistance gene to PM from *D. villosum*, was introgressed into common wheat using chromosome translocation, which was widely used in wheat production and became one of the most effective genetic loci introgressed into wheat from its wild species. Commercial wheat varieties carrying *Pm21* are widely applied in China, with an accumulative planting area of more than four million hectares [5]. In addition, *D. villosum* also contains many other excellent traits such as cold tolerance, strong tillering ability, dense spikes with many flowers, and high crude protein content in grain [6,7]. Currently, more than 300 *D. villosum* accessions were collected, and four of them were introgressed into wheat as additional lines, substitution lines, or translocation lines [8,9,10,11].

Chromosomal disproportionation in different *D. villosum* accessions can cause homologous chromosomes to be unpaired in their hybrids in a wheat background such as 6V#1 and 6V#2 [12]. Chromosome arms 6V#2S and 6V#4S can pair with each other only at a lower frequency in their F_1_ hybrids between 6V#2S·6AL and 6V#4S·6DL translocation lines by cytological observation [10]. In order to rule out the possible effect on the pairing behavior of the alien arms that might be caused by the different dynamics of 6AL and 6DL, it is necessary to investigate the pairing behavior between chromosomes 6V#2 and 6V#4 in the non-translocation state. Chromosome 6V#2 carries *Pm21*, which encodes a typical coiled-coil - nucleotide-binding site - leucine-rich repeat (CC–NBS–LRR) protein that confers broad-spectrum resistance to PM [5,13]. Chromosome 6V#4 carries *PmV*, which is considered an allele of *Pm21* according to data on the National Center for Biotechnology Information (NCBI) website, but its allelism needs to be confirmed. Pairing and exchange between the two chromosomes are prerequisites for the allelism test. PM resistance is the only phenotypic trait that can be tracked in a wheat background; most of the plants showed resistance in the F_2_ and testcross populations, but they cannot be distinguished from each other by phenotype.

A high collinear relationship between gramineous plant genomes was proven using comparative genomics strategies [14]. Specific markers to each chromosome of *D. villosum* were obtained using a comparative genomics method [10,15,16,17,18,19,20,21]. Some markers show polymorphisms in amplified length and presence or absence of specific amplified bands for the chromosomes of different *D. villosum* accessions. It should be possible to understand the affinity of the homologous chromosomes from different *D. villosum* accessions by investigating whether the exchange occurs among these markers in the F_2_ populations. At the same time, the conservation of marker ranks on the homoeologous chromosomes can be well defined by comparing the genetic linkage map of the alien chromosome with the physical map of its homoeologous chromosomes in wheat and barley. These results will provide some important information for understanding the phylogenetic status of *D. villosum* in the grass family and the selection of breeding strategies to induce homoeologous chromosomal translocations between alien species and wheat.

In this study, the pairing behavior of two alien chromosomes, 6V#2 and 6V#4, was investigated in the F_1_ hybrids derived from a cross between substitution lines 6V#2 (6A) and 6V#4 (6D) by genomic in situ hybridization (GISH). The genetic linkage map was drawn according to the exchange frequencies of the nine polymorphic PCR markers between the two alien chromosomes in the F_2_ populations. Furthermore, the physical maps of homologous sequences of the nine markers in wheat and barley were compared. Combined with the molecular marker-assisted selection (MAS) of wheat chromosomes 6A and 6D, three new types of substitution lines were identified, which laid a foundation for further screening of novel resistance to PM wheat–*D. villosum* translocation lines between the alien chromosomes and different wheat chromosomes in the sixth homoeologous group, as well as evaluating their genetic effects.

## 2. Results

### 2.1. Pairing Behavior of 6V#2 and 6V#4 Chromosomes in Their F_1_ Hybrids

**Figure 1:**
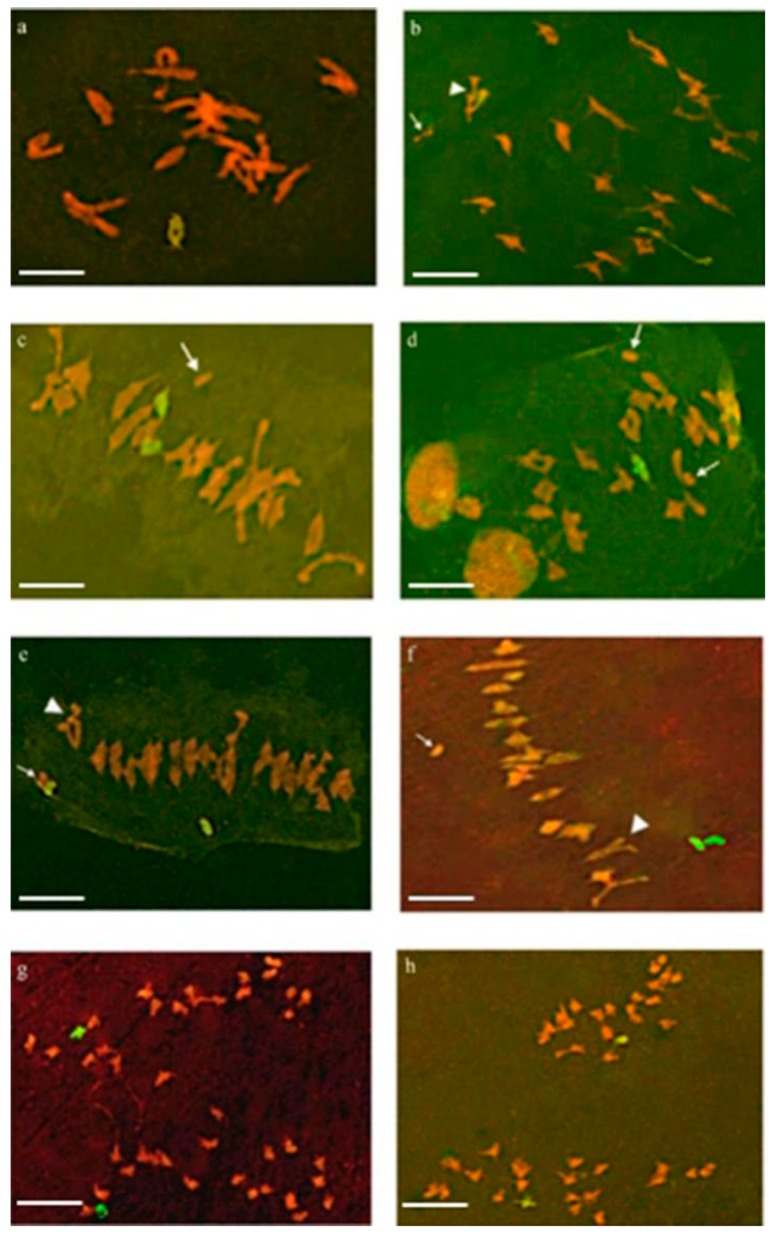
Chromosome pairing and separating behaviors in pollen mother cells (PMCs) of the F_1_ hybrids between 6V#2 (6A) and 6V#4 (6D) substitution lines as revealed by genomic in situ hybridization (GISH) analysis at meiosis. Red indicates wheat chromosomes counterstained with propidium iodide (PI); green indicates alien chromosomes. The triangular arrow indicates heteromorphic bivalent chromosomes, and the arrow indicates wheat univalent chromosomes. (**a**) Alien chromosomes paired with each other at diakinesis. (**b**) Alien chromosomes paired with each other to form bivalent rods at metaphase I. (**c**) Premature separation of alien chromosomes that formed bivalent rings. (**d**) Alien chromosomes paired with each other to form bivalent rings at metaphase I. (**e**,**f**) Unpaired univalent chromosomes 6V#2 and 6V#4. (**g**,**h**) Isolated homologous chromosomes 6V#2 and 6V#4 at anaphase I; wheat chromatin bridges can be seen. Bar = 10 μm.

In total, 107 pollen mother cells (PMCs) at meiosis in the F_1_ hybrids of 6V#2 (6A) and 6V#4 (6D) substitution lines were observed. Based on the observation of the green fluorescent signal, two alien chromosomes paired with each other at diakinesis (Figure 1a) and metaphase I (Figure 1b,c) in most of the PMCs. Here, 6V#2 paired with 6V#4 to form a rod bivalent (64.18% of the bivalent chromosomes) (Figure 1b) or a ring bivalent (35.82% of the bivalent chromosomes) (Figure 1c) in 67 PMCs (79.76%). A few of the alien bivalent rings separated in advance (Figure 1d). In 17 PMCs (20.24%), the alien chromosomes did not pair, and presented as two univalent chromosomes (Figure 1e,f). At anaphase I, the alien chromosomes were evenly pulled to the two poles in most PMCs (Figure 1g,h). At metaphase I, most of the PMCs showed a trivalent and a univalent wheat chromosome in the same cell (Figure 1b,e,f). Because 6A and 6D chromosomes lacked a pairing partner in the F_1_ hybrids, they should be present as two univalents, suggesting that there is a translocation involving 6D or 6A in wheat. At anaphase I, chromatin bridges were correspondingly observed in some PMCs (Figure 1g).

### 2.2. Detection of Plant Genotypes by Nine 6VS-Specific Molecular Markers in the F_2_ Populations

**Figure 2:**
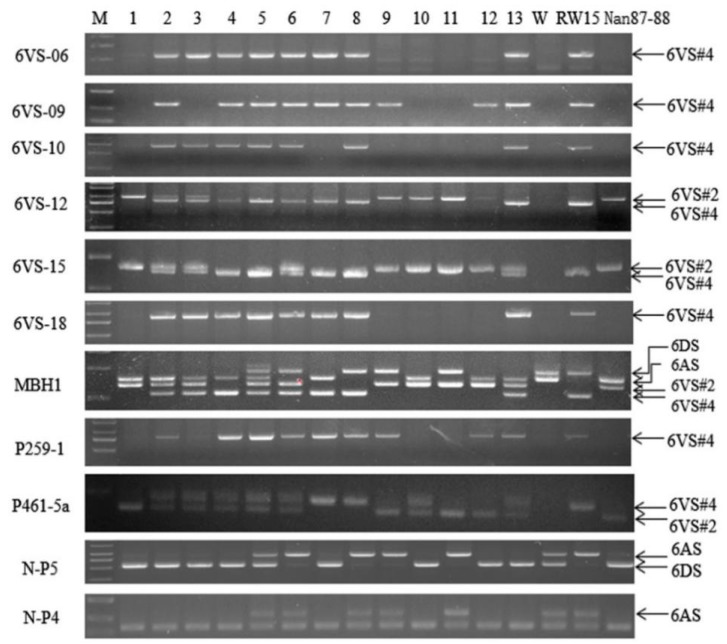
Amplification of portions of individual plants in F_2_ hybridization populations derived from two substitution lines with different markers. Samples 1–13 are individual F_2_ plants in 18GL101-11 from a cross of two substitution lines. M: DL2000+; W: Wan7107; RW15: substitution line 6VS#4 (6D); Nan87-88: substitution line 6VS#2 (6A).

A total of 323 individual plants were randomly selected from the F_2_ populations derived from the cross between the two substitution lines, and their genomic DNA samples were amplified by the nine 6V#2/6V#4-polymorphic PCR markers. The amplification of portions of the plants is shown in Figure 2; their amplified bands are listed in Table 1.

Among all selected plants, 83 plants had amplified bands identical to one of the parental types by the nine markers, including 30 with 6V#2- and 53 with 6V#4-specific bands (sample 1 and sample 8 in Figure 2 and Table 1). A total of 239 plants showed heterozygotic genotypes, which had both 6V#2- and 6V#4-specific bands in all or some of the codominant marker loci (samples 2–6, 10, and 13 in Figure 2 and Table 1), or recombinant genotypes with 6V#2-specific bands in some loci while having 6V#4-specific bands in other loci (samples 7, 9, and 12 in Figure 2 and Table 1). In one plant, neither 6V#2 nor 6V#4 chromosomes were detected.

A chi-square test was performed on the separation ratios of the nine markers in the F_2_ populations. The chi-square values of four dominant markers, 6VS-06, 6VS-10, 6VS-18, and P259-1, were all less than the *X^2^* value 6.635 of 3:1 at the 0.01 level (Table 2), consistent with the separation ratio of a Mendelian allele. The chi-square value of 6VS-09 was much larger than 6.635, indicating its deviation from the Mendelian ratio. The chi-square values of the codominant polymorphic marker 6VS-12 were higher than 9.210 at the 0.01 level (Table 2), indicating that the actual observed value was inconsistent with the theoretical value of 1:2:1. However, the chi-square values of MBH1, 6VS-15, and P461-5a were less than 9.210 at 0.01 level, which are consistent with the theoretical ratio of 1:2:1.

### 2.3. Construction of the 6VS Genetic Linkage Map and Collinearity Analysis

**Figure 3:**
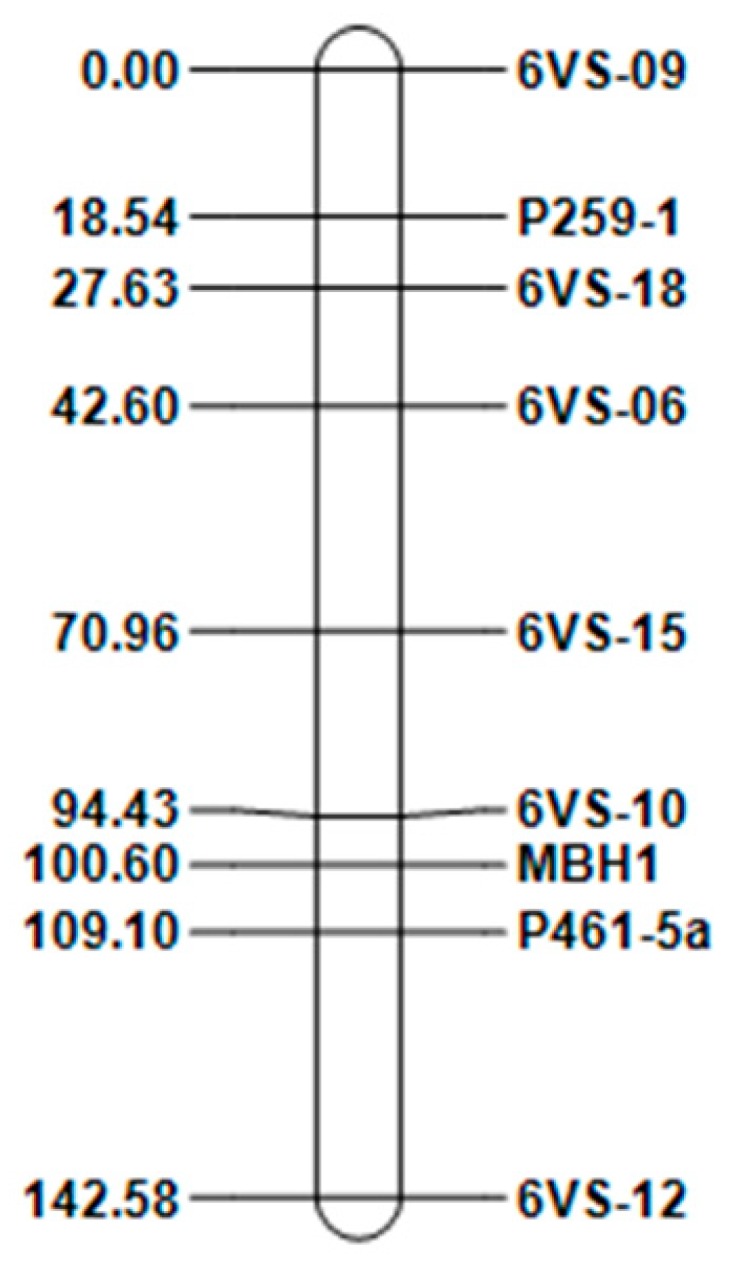
Genetic linkage map constructed based on the exchange frequencies of nine markers in the F_2_ populations of two substitution lines.

To further determine the linear order of the nine markers on chromosome 6VS, the mapping software QTL IciMapping was used with an LOD value of 3.0. The genetic linkage map was drawn with a genetic recombination rate as the distance between the markers. The map spans a total distance of 142.58 cM (Figure 3).

**Figure 4:**
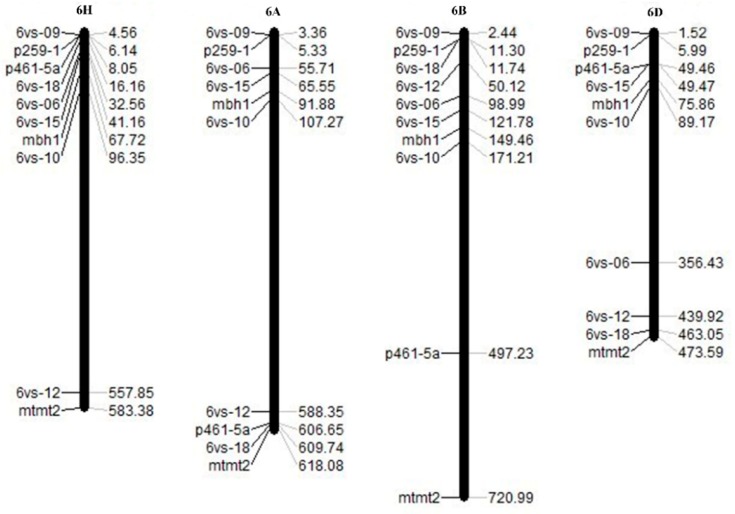
Physical maps of wheat 6A, 6B, and 6D and barley 6H based on homologous sequences of nine markers on 6VS.

Sequences of the nine markers were BLASTed on the website http://plants.ensembl.org/index.html to obtain the physical location information of the homologous sequences of the homoeologous group 6 of wheat and barley, and physical maps were drawn. The distribution of the nine markers on chromosomes of wheat homoeologous group 6 and barley 6H is shown in Figure 4. Compared with the physical maps of barley 6H and wheat 6A, 6B, and 6D, out of the nine homologous sequences, there were eight located on 6HS and 6BS, and six on 6AS and 6DS, while the others were found on the long arms of the corresponding chromosomes. The genetic map of the nine markers had the highest similarity in order on 6H and 6B, while the similarity in order on the three wheat chromosomes was 6B > 6A > 6D.

### 2.4. Development of 6A- and 6D-Specific Molecular Markers and Detection of 6A and 6D Chromosomes in the F_2_ Populations

**Figure 5:**
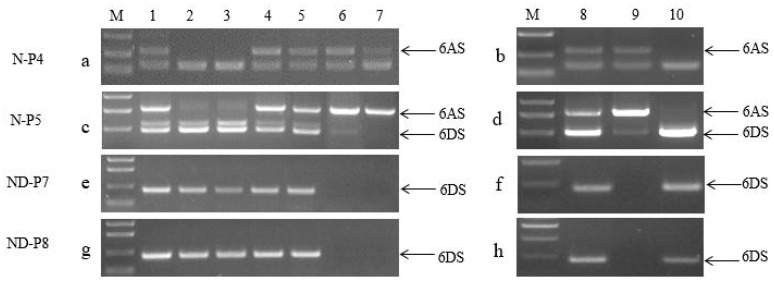
Amplification patterns of 6AS-/6DS-specific molecular markers in wheat, nulli-tetrasomic lines of 6A, 6B, and 6D, and translocation lines. (**a,b**) Samples were amplified by marker N-P4. (**c,d**) samples were amplified by marker N-P5. (**e,f**) samples were amplified by marker ND-P7. (**g,h**) samples were amplified by marker ND-P8. M: DL5000; 1 and 8, Wan7107; 2, CSN6AT6B; 3, CSN6AT6D; 4, CSN6BT6A; 5, CSN6BT6D; 6, CSN6DT6A; 7, CSN6DT6B; 9, 6V#4S·6DL translocation line Pm97033; 10, 6V#2S·6AL translocation line Nanyi.

For developing 6A- and 6D-specific molecular markers, 197 pairs of primers were designed. One pair of primers, N-P4 (Table 3), was amplified in CSN6BT6A, CSN6BT6D, CSN6DT6A, and CSN6DT6B, but not CSN6AT6B and CSN6AT6D (Figure 5a), indicating that it is a 6A-specific marker. Another pair of primers, N-P5 (Table 3), was amplified two bands in CSN6BT6A and CSN6BT6D, the larger one corresponding to the band amplified in CSN6AT6B and CSN6AT6D, and the smaller one corresponding to the band amplified in CSN6DT6A and CSN6DT6B (Figure 5c); therefore, it should be a codominant polymorphic marker of 6A and 6D. The two translocation lines 6V#2S·6AL and 6V#4S·6DL, lacking 6AS and 6DS, respectively, were amplified by N-P4 and N-P5. The results showed that the marker N-P4 could not amplify a band in the translocation line 6V#2S·6AL, but it could amplify a band in the translocation line 6V#4S·6DL (Figure 5b), indicating that N-P4 was a specific marker to 6AS. N-P5 amplified two bands in a wheat line Wan7107, corresponding to the bands in the two translocation lines (Figure 5d), which showed that N-P5 was a real polymorphic marker of 6AS and 6DS. The other two 6D-specific molecular markers ND-P7 and ND-P8 were developed using a similar method, and their amplifications are shown in Figure 5e–h.

According to a previous report [23], MBH1 can specifically amplify 6V#2S/6V#4S/6AS/6DS chromosomes simultaneously. We carefully investigated the amplification bands of MBH1 in the F_2_ populations, and further verified them with the markers N-P4 and N-P5 developed in this study. Based on the specific amplified bands of 6A and 6D, the transmission rate of 6D was higher than that of 6A in the F_2_ populations (Table 4).

### 2.5. Development of Pm21 and Its Allele-Specific Molecular Markers and Detection of Both Genes in the Testcross Populations

**Figure 6:**
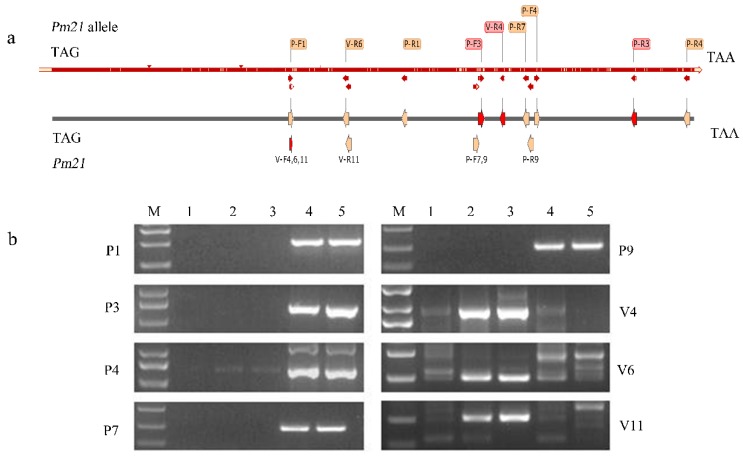
*Pm21* and its allele-specific markers and their chromosomal distribution. (**a**) The schematic diagram of the distribution of all primers for the *Pm21* gene and its alleles. The *Pm21* gene and its alleles were amplified with primers (P3 and V4) marked by red arrows in this experiment. (**b**) Development of molecular markers of the *Pm21* gene and its alleles. M: DL5000; 1: Wan7107; 2: translocation line 6V#4S·6DL; 3: Hv-s (6V#4); 4: translocation line 6V#4S·6AL; 5: CMM (6V#2).

In order to develop specific markers to detect the PM resistance gene *Pm21* on 6V#2 and its allele on the 6V#4 chromosome in a common wheat background, a multiple sequence alignment was conducted between the two genes and their homologous sequences in wheat. Primers were designed among the polymorphic sites. The schematic diagram of the distribution of primers on both alien genes is shown in Figure 6a. Primers V4, V6, and V11 are linked with the *Pm21* allele, while primers P1, P3, P4, P7, and P9 are linked with *Pm21*; their amplifications are shown in Figure 6b.

**Figure 7:**
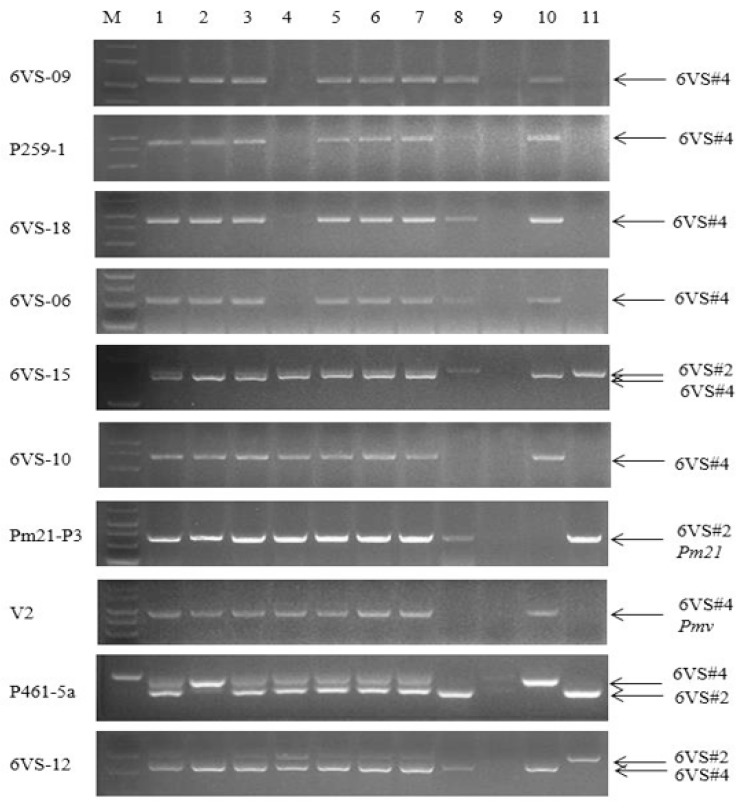
Amplification of some individuals in the testcross generations. Samples 1–8 are individuals from a testcross generation. M: DL5000; 9: Wan7107; 10: translocation line 6V#4S·6DL; 11: translocation line 6V#2S·6AL.

The newly developed primers were used to trace the alien chromosomes in the testcross generations with 482 individuals. *Pm21* or its allele-specific markers were amplified in most of the plants. However, there were 16 plants where no alien-specific bands were amplified, of which seven showed susceptibility and nine showed resistance to PM. Moreover, there were seven other plants where both *Pm21* and its allele were amplified. In order to confirm the results, eight additional 6V#2/6V#4-polymorphic PCR markers were used to amplify bands in the individuals that contain both genes. The results indicated that 6V#2- and 6V#4-specific bands were also simultaneously amplified by three co-dominant markers except in one plant, which only had bands specific to the 6V#4 chromosome, suggesting that it was a recombinant of *Pm21* and its allele or a heterozygote of two alien chromosomes (sample 2 in Figure 7).

### 2.6. Identification of New Genotypes

According to the amplified bands by exogenous chromosome-specific markers and A and D chromosome-specific markers in group 6, we found that, in addition to the parental substitution types 6V#2 (6A) (sample 1 in Figure 2 and Table 1) and 6V#4 (6D) (sample 8 in Figure 2 and Table 1), there were three new types of substitution lines of 6V#2 (6D) (sample 11 in Figure 2 and Table 1), 6V#4 (6A) (sample 7 in Figure 2 and Table 1), and 6D or 6A chromosomes substituted by 6V#2#4 recombinant chromosome (samples 9 and 12 in Figure 2 and Table 1) in the F_2_ populations.

## 3. Discussion

### 3.1. Distribution of Amplified Sequences on Chromosomes and Their Separation Ratios in Hybrid Progenies

In this study, nine molecular markers were used to detect exchange events between the two alien chromosomes 6V#2 and 6V#4 in the F_2_ populations derived from the two alien substitution lines. The separation ratio of the nine markers was investigated based on the chi-square test. Among them, seven markers segregated consistently with the theoretical ratios 3:1 or 1:2:1 of Mendel’s separation law at the 0.01 level, suggesting that the alien chromosomes had regular separating behavior, which is consistent with cytological observations of F_1_ hybrids.

However, the separation ratios of two markers, 6VS-09 and 6VS-12, did not fit the theoretical ratios. Different competitiveness of gametes or genetic disorder in distant hybridization was suggested as the cause for distorted segregation [18,19,24,25]. For PCR-based molecular markers, we believe that the separation ratio might be closely related to the copy number and distribution mode of primer amplification regions on the genome or chromosome, and the sequences of primers.

Due to lacking genomic sequence information of *D. villosum*, the copy number of the amplified regions on 6V and the distance between different copies are not clear. However, BLAST performed on the sequences of the nine aforementioned molecular markers showed considerable variation in the copies of the homologous sequences on wheat chromosomes 6A, 6B, and 6D, and barley chromosome 6H and their chromosomal distributions. Therefore, if these markers have more than one copy of primer-matched binding sequences, and exchange occurs at the same time between these copies, it may affect the separation ratio of the molecular markers in the segregation populations. In addition, other factors might lead to significant differences between the actual statistical results and the theoretical results, including distribution differences of homologous sequences on the two alien chromosomes, and competitive amplification caused by the complexity of some primers binding to DNA template (for example, when some primers amplified the mixed DNA samples of translocation lines 6V#2S·6AL and 6V#4S·6DL, occasionally, priority was given to the short target fragment).

### 3.2. Two Alien Chromosomes 6V#2 and 6V#4 Pair and Exchange in Their Hybrids

Both 6V#2S and 6V#4S are known to contain powdery mildew (PM) resistance genes, which is the only phenotypic trait that can be tracked in a wheat background, but they cannot be distinguished by phenotype from each other. Therefore, it is impossible to determine the existence of exchange and recombination by phenotype in F_2_ hybrids. In addition, the feasibility of the allelic test can only be confirmed by verifying that exchange and recombination events can occur.

Some alien chromosomes cannot be properly paired and exchanged in a wheat background, on account of the large genetic differences between homologous chromosomes from different *D. villosum* sources. For example, Qi et al. [12] selected 40 restriction fragment length polymorphism (RFLP) probes specific to the sixth homoeologous chromosomes of wheat to analyze disomic substitution lines 6V#2 (6A) and 6V#1 (6A), for which 25 probes could detect the differences between 6V#2 and 6V#1. Using a cytological technique, the authors observed that chromosomes 6V#2 and 6V#1 did not pair, indicating that they had significant structural differences. According to our previous research, chromosome-pairing frequency of 6V#2S and 6V#4S in the F_1_ hybrids between translocation lines 6V#2S·6AL and 6V#4S·6DL was 18.9% [26], and, in most cases, they were not paired. This might be related to different structures of the two alien chromosome arms and the kinetic difference during the meiosis process of each translocated wheat chromosome.

In this study, two alien substitution lines, 6V#2 (6A) and 6V#4 (6D) were hybridized to construct F_2_ segregating populations. Whether the two alien chromosomes 6V#2 and 6V#4 can normally pair and exchange without the involvement of translocation between alien and wheat chromosomes was investigated. Using the nine markers to detect the F_2_ generations, we found that non-exchanged parental type was 25.70%, exchange type was 73.99%, and wheat type without alien chromosomes was 0.31%, indicating that pairing and exchanging between the two alien chromosomes occurred at a rational frequency. However, it was observed that the percentage of the alien bivalent rod was much higher than that of the alien bivalent ring formed at meiosis in the F_1_ hybrids, indicating that the pairing between 6V#2S and 6V#4S is still limited. Therefore, the allelism test should be carried out in a larger populations.

The type of gametes formed in the F_1_ hybrids can be detected in the testcross populations. Theoretically, each individual in the testcross populations should contain only one alien chromosome. According to our results, out of 482 individuals, two exogenous chromosomes, 6V#2 and 6V#4, were simultaneously amplified in seven individuals by *pm21* and its allele-specific markers and three other molecular markers, 6VS-12, 6VS-15, and P461-5a. Moreover, seven PM-susceptible individuals without alien chromosomes were detected as well. These results suggested that nearly 1.5% of the alien homologous chromosomes or the sister chromosomes did not separate, and both moved to the same pole during meiosis anaphase I or II, resulting in the other pole lacking the corresponding alien chromosomes, thus forming the types we detected. This can also be supported by the fact that 20.24% of alien chromosomes are not paired during the meiosis of F_1_ hybrids. In the testcross populations, we also detected nine PM-resistant plants lacking alien chromosome-specific markers. A possible explanation for this might be other unknown PM resistance gene(s) in the wheat genome.

### 3.3. Screening of Molecular Markers for Novel Alien Substitution Lines

At present, substitution lines are mainly derived from five methods including spontaneous substitution, monosomic substitution, telosomic substitution, nullisomic backcrossing, and tissue culture [27,28,29,30]. In theory, the homoeologous chromosomes of three wheat subgenomes can be replaced with alien chromosomes, forming three different types of alien substitution lines. The type of alien substitution lines might be related to the developing technique. Recently, we identified 6V#4 (6D) alien substitution lines from the progeny of *T. durum–D. villosum* amphidiploids crossed and backcrossed with common wheat (unpublished). In this case, genomes A and B have a homologous pairing partner during meiosis in F_1_ and backcrossing generations, and they can normally separate at anaphase I. Genomes V and D lack their homologous partner chromosomes and, thus, D genome chromosomes are more easily replaced by V chromosomes.

**Figure 8:**
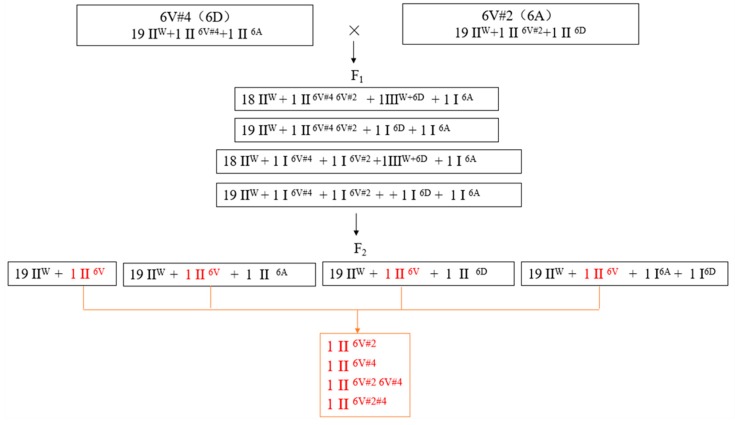
Development of new exchange types in this study. W indicates wheat chromosomes; 6V indicates alien chromosomes. There are four possible combinations of II^6V^ in F_2_ shown in red, which are II^6V#2^, II^6V#4^, II^6V#26V#4^ (heterozygote), and II^6V#2#4^ (recombinant).

In this study, two different substitution lines, 6V#4 (6D) and 6V#2 (6A), were used to hybridize; theoretically, in the F_1_ hybrids, 6V#2 and 6V#4, as homologous chromosomes, can be paired and exchanged with each other to form new recombinant chromosomes if they have high affinity without significant structural differences. In contrast, chromosomes 6A and 6D, due to a lack of homologous chromosomes, became two univalents at meiosis metaphase I. In addition, the two univalents separated randomly at anaphase I, and were independently assorted in the F_2_ offspring, which provided a chance for the formation of new substitution types (Figure 8). Unexpectedly, a trivalent chromosome and a univalent chromosome instead of two univalent chromosomes were observed at metaphase I, suggesting that a 6A or 6D chromosome was involved in the trivalent. At the same time, the frequency of chromosome 6D in the F_2_ generation was higher than that of chromosome 6A. Considering that 6A or 6D was involved in the trivalent at meiosis metaphase I, 6D should be the target participating in the translocation event. Using the MAS technique, we obtained three new types of substitution lines and new alien chromosome-exchange types in F_2_ and F_3_, which lays the foundation for further genetic evaluation, such as studying the differences of genetic effects between 6V#4 (6A) and 6V#4 (6D) or 6V#2 (6A) and 6V#2 (6D).

### 3.4. Importance of Genetic Linkage Map for Genera That Lack Genome Sequence Information

Genetic linkage maps provide breeders with the order of markers and information of target genes on the chromosomes [31,32,33,34,35]. Molecular markers are suitable for constructing genetic linkage maps. These molecular markers not only reflect the genetic diversity at the DNA level, but also show high polymorphism, and co-dominance features that can identify homozygous and heterozygous genotypes [36]. The basic principle of constructing genetic linkage maps is generated from eukaryotes undergoing meiosis, in which the chromosomes are recombined and exchanged, and the exchanging and recombination rates are also affected by the relative distance between any two sites on chromosomes. Qi et al. [37] mapped more than 16,000 expressed sequence tag (EST) loci to specific physical intervals on different wheat chromosomes. These EST sequences and localization provided information for constructing a genetic linkage map of target genes.

At present, the genome databases of wheat, barley, and other cereal crops are gradually improving with the rapid development and cost reduction of sequencing technology [38,39,40,41,42], which provides great convenience for the study of wild wheat species without genomic information. The *ph1b* gene can induce pairing and exchange between alien chromosomes and their homoeologous chromosomes in wheat backgrounds [43,44]. The paired homoeologous chromosomes need to have high collinearity, and structural variation of chromosomes including the inversion of fragments may influence the chromosomes pairing and exchanging. In this study, a genetic linkage map was constructed using nine markers, and the order of the markers on the genetic linkage map 6V was nearly consistent with those on barley 6H and wheat 6B, followed by 6A and 6D. The quantity of markers used in this study was limited; thus, we need to develop additional molecular markers and increase the density of markers on chromosome 6VS. Increasing marker density is necessary for making more accurate maps and better evaluating the conservation of the linear ordering of the sequences on alien chromosomes with those of their wheat homoeologous chromosomes. Such studies are of great significance for investigating the phylogenetic relationship between *D. villosum* and other cereal crops, transferring beneficial genes into wheat, and obtaining an ideal genetic compensation effect in translocation materials.

## 4. Materials and Methods

### 4.1. Plant Materials

*D. villosum* accession *D.v#2* from Cambridge Botanical Garden in England and its derived T6V#2S·6AL translocation line Nanyi, and 6V#2(6A) alien substitution line Nan87-88 were provided by Peidu Chen at Nanjing Agricultural University, China. Chinese Spring group 6 nulli-tetrasomic stocks CSN6AT6B, CSN6AT6D, CSN6BT6A, CSN6BT6D, CSN6DT6A, and CSN6DT6B were provided by Dr. Steven Xu (USDA-ARS, Northern Crop Science Laboratory, USA) and preserved at the Institute of Crop Science (ICS), Chinese Academy of Agricultural Sciences (CAAS). *D. villosum* accession No. 1026 from Russia and its derived T6V#4S·6DL translocation line Pm97033, 6V#4(6D) alien substitution line RW15, and wheat cultivar Wan7107 were provided by professor Chen Xiao at ICS, CAAS. The F_1_ populations were derived from a cross between Nan87-88 and RW15, and the F_2_ populations were obtained by self-crossing of the F_1_ plants. Testcross generations were obtained from a cross between F_1_ populations and wheat cultivar Wan7107. The phenotype of wheat powdery mildew resistance was evaluated at the seedling stage and mature plant stage.

### 4.2. Molecular Markers

Nine 6V-specific molecular markers developed previously in our laboratory [10,22] and by Bie et al. [23] were used in the present study. Four 6AS- and 6DS-specific markers were developed for this study as follows: sequences of chromosome 6A/6B/6D in Fasta format were downloaded from http://plants.ensembl.org/Triticum_aestivum/Info/Index. The files were opened in SnapGene software (https://www.snapgene.com/), and a fragment from one chromosome with a certain size at the exon was selected. BLAST analyses were conducted using the Ensembl website, and the sequences with the highest identity to this fragment on the short chromosome arms of the other two genomes in the sixth homoeologous group were selected. Primers were designed based on the region showing the polymorphic sequences among the three genomes using Primer premier 6.0 software (http://www.premierbiosoft.com/primerdesign/), and synthesized by Sangon Biotech company (Shanghai, China).

### 4.3. DNA Extraction and PCR Amplification Conditions

Genomic DNA was extracted from 1 g of the leaves of two-month-old seedlings using Nuclear Plant Genomic DNA Kit (CW Bio Inc., Beijing, China). The DNA pellet was dissolved into a concentration of 100 ng·μL^−1^ in sterile water, and stored at −20 °C. PCR was carried out in a 15-µL reaction volume consisting of 100 ng of template DNA, 0.3 µL of each primer (10 µmol·L^−1^), 5.9 µL of water, and 7.5 µL of 2× Taq Master Mix (containing Mg^2+^ and dNTPs, Vazyme Biotech Co., Ltd., Nanjing, China). PCR amplification was performed in a Biometra Professional thermal cycler (Göttingen, Germany) with an initial denaturation at 95 °C for 5 min, 35 cycles of 20 s at 95 °C, 30 s at 54–60°C (annealing temperatures varied with the primer), 40–60 s (extension times varied with the product size) at 72 °C, and a final extension of 8 min at 72 °C. PCR products were separated on 1.5% agarose gels with a standard TAE buffer (40 mM Tris, 20 mM acetic acid, 1 mM EDTA) and visualized in a gel doc system (Bio-Rad, Hercules, CA, USA) after Genecolour II staining.

### 4.4. Construction of a Linkage Map and Comparison Maps

In this study, 323 plants in the aforementioned F_2_ populations were randomly selected and analyzed using the nine polymorphic molecular markers. QTL IciMapping software [45] (http://www.isbreeding.net/) was used to draw a genetic linkage map.

The sequences of the nine 6VS-specific markers were BLASTed on the website http://plants.ensembl.org/index.html. According to the physical position information of the homologous sequences on wheat 6A, 6B, 6D and barley 6H, the physical maps were drawn and compared.

### 4.5. Cytological Procedures

Anthers at meiosis stages were fixed in Carnoy’s Fluid for 24 h and then transferred to 70% ethanol and stored at −20 °C. Before cytological preparation, the anthers were washed with water three times, then put into 1× (A + B) solution (0.1 M citric acid + 0.1 M tri-sodium citrate) for 11 min, and transferred to enzymatic hydrolysate (2% cellulase R-10 + 2% pectinase Y-23, Japan) at 37 °C for 6.5 min. The reaction was terminated by adding 1× (A + B) solution. Anthers were put on slides, and one drop of 45% glacial acetic acid was added; the anther was lightly mashed with a dissecting needle, and then covered with a coverslip and pressed vertically with the thumb. The slide was then frozen in liquid nitrogen and stored at −20 °C after removing the coverslip.

Genomic in situ hybridization was carried out according to the method introduced by Wei et al. [46]. DIG-Nick Translation Mix Kit (Roche, Mannheim, Germany) was used for probe labeling, the total genomic DNA of *D. villosum* was labeled to use as a probe, and Chinese Spring genomic DNA was included as blocking DNA. Hybridization signals were identified by using a fluorescein isothiocyanate (FITC)-conjugated Anti-Digoxigenin Fluorescein Fab Fragments Kit (Roche, Mannheim, Germany). Observation of chromosome pairing configuration was carried out with a fluorescence microscope (BX51, Olympus Co., Ltd., Tokyo, Japan).

## 5. Conclusions

Firstly, 6V#2 and 6V#4, chromosomes from different *Dasypyrum villosum* accessions, were found to pair and exchange. This was confirmed by cytological observation in their F_1_ hybrids, and by analyzing nine molecular markers in their F_2_ populations, which allows genes controlling desirable traits from different alien chromosomes to be pyramided, and provides important information for the alien allelism test of some vital genes. Secondly, the genetic linkage map of nine markers on 6VS was compared with the physical maps of wheat and barley based on homologous sequences of the markers, which showed that the conservation of sequence order compared to 6V was 6H, 6B > 6A > 6D. Finally, three wheat–*D. villosum* substitutions 6V#4 (6A), 6V#2 (6D), and the alien recombinants 6V#2#4 (6A/6D) with novel resistance to powdery mildew were selected using 6V-, 6A-, and 6D-specific molecular markers.

## Figures and Tables

**Table 1:** Genotypes of portions of the F_2_ plants from the crossing of two substitution lines amplified by different markers.

Materials	1	2	3	4	5	6	7	8	9	10	11	12	13	Wan 7107	RW15	Nan 87–88
Markers																
6VS-06	-	4	4	4	4	4	4	4	-	-	-	-	4	-	4	-
6VS-09	-	4	-	4	4	4	4	4	4	-	-	4	4	-	4	-
6VS-10	-	4	4	4	4	4	-	4	-	-	-	-	4	-	4	-
6VS-12	2	2, 4	2, 4	4	4	4	4	4	2	2	2	-	4	-	4	2
6VS-15	2	2, 4	2, 4	4	4	2, 4	4	4	2	2	2	2	2, 4	-	4	2
6VS-18	-	4	4	4	4	4	4	4	-	-	-	-	4	-	4	-
MBH1	2	2, 4	2, 4	4	2, 4	2, 4	4	4	2	2	2	2	2, 4	-	4	2
P259-1	-	4	-	4	4	4	4	4	4	-	-	4	4	-	4	-
P461-5a	2	2, 4	2, 4	2, 4	2, 4	2, 4	4	4	2	2,4	2	2	2, 4	-	4	2
N-P4	-	-	-	-	6A	6A	-	6A	6A	-	6A	-	-	6A	6A	-
N-P5	6D	6D	6D	6D	6A, 6D	-	6D	-	-	6D	-	6D	6D	6A, 6D	6A	6D

Samples 1–13 are 13 individual F_2_ plants of 18GL101-11 from the crossing of two substitution lines; “2” stands for specific band amplified from alien chromosome 6V#2S; “4” stands for specific band amplified from alien chromosome 6V#4S; “2,4” stands for specific bands amplified from alien chromosome 6V#2S and 6V#4S; “6A” stands for specific band amplified from wheat chromosome 6A; “6D” stands for specific band amplified from wheat chromosome 6D; “6A,6D” stands for specific bands amplified from wheat chromosomes 6A and 6D; “-” indicates the plant without the corresponding amplified band.

**Table 2:** Chi-square test with nine 6V#2S/6V#4S specific markers in the F_2_ populations derived from the cross of two alien substitution lines.

Markers	Total	#2^+^	#4^+^	#2^+^#4^+^	Chi-Square Value *X*^2^_3:1_ = 6.635, *X*^2^_1:2:1_ = 9.210, *p* = 0.01
6VS-06	323	-	233	-	1.41 < *X*^2^_3:1_
6VS-09	323	-	198	-	32.33 > *X*^2^_3:1_
6VS-10	323	-	228	-	3.35 < *X*^2^_3:1_
6VS-18	323	-	229	-	2.90 < *X*^2^_3:1_
P259-1	323	-	236	-	0.64 < *X*^2^_3:1_
MBH1	316	84	79	153	0.47 < *X*^2^_1:2:1_
6VS-12	307	55	103	149	15.27 > *X*^2^_1:2:1_
6VS-15	125	36	30	59	0.97 < *X*^2^_1:2:1_
P461-5a	308	77	94	137	5.63 < *X*^2^_1:2:1_

**Table 3:** The molecular markers used in this study.

Primer	Forward Sequence (5′→3′)	Reverse Sequence (5′→3′)	Specific Type	Reference
N-P4	TTAAGAATGTAAGATCGTTGACCCGTAGAC	GGACTGTGACTTGTGAGCATGATGT	6AS	Present study
N-P5	GCGACCTGTTAGAATGCTATTACGATTAC	ATGCTACTCTACCGATGCTTTGAACC	6AS, 6DS	Present study
ND-P7	AACTCTAAGCTCCGCATCATCAATCAT	CTGCTGCCTCATCCAGTTACCAAG	6DS	Present study
ND-P8	GCAACTCTAAGCTCCGCATCATCA	CTGCTGCCTCATCCAGTTACCAAG	6DS	Present study
P1	AGAGTATTTGGTTCCGGATATG	TTTCTGCACACTTGCTGAGGAT	6V#2 (*Pm21*)	Present study
P3	CATACGGAATAGATTTTCCTACCGAAT	TAGCCCTATCTGAAACTGCATGTC	6V#2 (*Pm21*)	Present study
P4	AGTCTGAGGGAGCTGAGGCTTTACA	GACCACATTCATAGAACTGAGGGGAA	6V#2 (*Pm21*)	Present study
P7	GTATGTCAAGGTTTCTGCTTCATACGG	ATATCCTCTAGCGAGATGTGCTCCATA	6V#2 (*Pm21*)	Present study
P9	GTATGTCAAGGTTTCTGCTTCATACGG	AGCTCGCCAAGGCCATTAATATCC	6V#2 (*Pm21*)	Present study
V4	TTTGGTTCCGGACCCTGCCC	GACAACCGTGGCAAGCAGACAA	6V#4 (*Pm21* allele)	Present study
V6	TTTGGTTCCGGACCCTGCCC	ACATGGACGGAGATGAAGAGGAAGAT	6V#4 (*Pm21* allele)	Present study
V11	TTTGGTTCCGGACCCTGCCC	AGTACAGGAGACATGGACGGAGATG	6V#4 (*Pm21* allele)	Present study
6VS-06	GACAGGCAGCTATGAGGC	AATCGTCGTTTGGAGTGG	6V#4S	[10]
6VS-09	GTAAGAACAAGAGGCTAAACAG	CCAGATGACGGTTATTACATAG	6V#4S	[10]
6VS-10	GCCATAAGTGACGCTGAT	GCATCCTGTGAAGTTGTTG	6V#4S	[10]
6VS-12	TGTTGCCTCTCCTCATCA	ATTGCTGTCCGCTCATAC	6V#4S, 6V#2S	[10]
6VS-15	AGGACCATACATTCACAGAG	TTCCATGAGCAGATTAGCA	6V#4S, 6V#2S	[10]
6VS-18	AGCCAGTAAGATTCCGTATG	TCTAACCTTCCTCACAACAC	6V#4S	[10]
P461-5a	GCGTCATCCGCGCCCGTCAGGT	GAGTGCTAATGATAGATGTG	6V#4S, 6V#2S	[22]
P259-1	CGTGATTCAGGAAATGCGATAC	TTGCGCCGCCATGTTAG	6V#4S	[22]
MBH1	GCCATTATAGTCAAGAGTGCACTAGCTGT	AGCTCCTCTCGTTCTCCAATGCT	6AS, 6DS, 6V#4S, 6V#2S	[23]

**Table 4:** Frequencies of chromosomes 6A and 6D in the F_2_ populations of two alien substitution lines.

Total Plants Investigated	6A^+^6D^−^	6A^−^6D^+^	6A^+^6D^+^	6A^−^6D^−^
319	86	121	97	15
100%	26.96%	37.93%	30.41%	4.70%

## Abbreviations

GISH	genomic in situ hybridization
MAS	marker-assisted selection
PMCs	pollen mother cells
FITC	fluorescein isothiocyanate
PCR	polymerase chain reaction
PM	powdery mildew

## References

[1] Grądzielewska A. (2006). The genus Dasypyrum-part 2. *Dasypyrum villosum*-a wild species used in wheat improvement. Euphytica.

[2] Pace C.D., Snidaro D., Ciaffi M., Vittori D., Ciofo A., Cenci A., Tanzarella O.A., Qualset C.O., Scarascia Mugnozza G.T. (2011). Introgression of *Dasypyrum villosum* chromatin into common wheat improves grain protein quality. Euphytica.

[3] Qi L., Friebe B., Zhang P., Gill B.S. (2007). Homoeologous recombination, chromosome engineering and crop improvement. Chromosome Res..

[4] Li K., Hegarty J., Zhang C., Wan A., Wu J., Guedira G.B., Chen X., Muñoz-Amatriaín M., Fu D., Dubcovsky J. (2016). Fine mapping of barley locusRps6conferring resistance to wheat stripe rust. Theor. Appl. Genet..

[5] Xing L., Hu P., Liu J., Witek K., Zhou S., Xu J., Zhou W., Gao L., Huang Z., Zhang R. (2018). *Pm21* from *Haynaldia villosa* Encodes a CC-NBS-LRR that Confers Powdery Mildew Resistance in Wheat. Mol. Plant..

[6] Blanco A., Resta P., Simeone R., Parmar S., Shewry P.R., Sabelli P., Lafiandra D. (1991). Chromosomal location of seed storage protein genes in the genome of *Dasypyrum villosum* (L.) Candargy. Theor. Appl. Genet..

[7] Chen P., Liu D. (1982). Cytogenetic studies of hybrid progenies between triticum aestivum and haynaldia villosa. J. Nanjing Agric. Univ..

[8] Blanco A., Simeone R., Resta P. (1987). The addition of *Dasypyrum villosum* (L.) Candargy chromosomes to durum wheat (*Triticum durum* Desf.). Theor. Appl. Genet..

[9] Li G., Zhao J., Li D., Yang E., Huang Y., Liu C., Yang Z. (2014). A Novel Wheat-*Dasypyrum breviaristatum* Substitution Line with Stripe Rust Resistance. Cytogenet. Genome Res..

[10] Li S., Lin Z., Liu C., Wang K., Du L., Ye X. (2017). Development and comparative genomic mapping of *Dasypyrum villosum* 6V#4S-specific PCR markers using transcriptome data. Theor. Appl. Genet..

[11] Li S., Wang J., Wang K., Chen J., Wang K., Du L., Ni Z., Lin Z., Ye X. (2019). Development of PCR markers specific to *Dasypyrum villosum* genome based on transcriptome data and their application in breeding *Triticum aestivum-D. villosum*#4 alien chromosome lines. BMC Genom..

[12] Qi L., Wang S., Chen P., Liu D., Gill B.S. (1998). Identification and physical mapping of three *Haynaldia villosa* chromosome-6V deletion lines. Theor. Appl. Genet..

[13] He H., Zhu S., Zhao R., Jiang Z., Ji Y., Ji J., Qiu D., Li H., Bie T. (2018). *Pm21* encoding a typical CC-NBS-LRR protein, confers broad-spectrum resistance to wheat powdery mildew disease. Mol. Plant.

[14] Liu Z., Zhu J., Hua W., Yang Z., Sun Q., Liu Z. (2011). Comparative Genomics Analysis and Constructing EST Markers Linkage Map of Powdery Mildew Resistance Gene *pm42* in Wheat. Acta Agron. Sin..

[15] Ando K., Krishnan V., Rynearson S., Rouse M.N., Danilova T., Friebe B., See D., Pumphrey M.O. (2019). Introgression of a Novel Ug99-Effective Stem Rust Resistance Gene into Wheat and Development of *Dasypyrum villosum* Chromosome-Specific Markers via Genotyping-by-Sequencing (GBS). Plant Dis..

[16] Zhang R., Zhang M., Wang X., Chen P. (2014). Introduction of chromosome segment carrying the seed storage protein genes from chromosome 1V of *Dasypyrum villosum* showed positive effect on bread-making quality of common wheat. Theor. Appl. Genet..

[17] Zhang R., Hou F., Feng Y., Zhang W., Zhang M., Chen P. (2015). Characterization of a *Triticum aestivum-Dasypyrum villosum* T2VS·2DL translocation line expressing a longer spike and more kernels traits. Theor. Appl. Genet..

[18] Zhang J., jiang Y., Wang Y., Guo Y., Long H., Deng G., Chen Q., Xuan P. (2018). Molecular markers and cytogenetics to characterize a wheat-*Dasypyrum villosum* 3V (3D) substitution line conferring resistance to stripe rust. PLoS ONE.

[19] Wang H., Dai K., Xiao J., Yuan C., Zhao R., Doležel J., Wu Y., Cao A., Chen P., Zhang S. (2017). Development of intron targeting (IT) markers specific for chromosome arm 4VS of *Haynaldia villosa* by chromosome sorting and next-generation sequencing. BMC Genom..

[20] Zhang R., Sun B., Chen J., Cao A., Xing L., Feng Y., Lan C., Chen P. (2016). Pm55, a developmental-stage and tissue-specific powdery mildew resistance gene introgressed from *Dasypyrum villosum* into common wheat. Theor. Appl. Genet..

[21] Cao Y., Cao A., Wang X., Chen P. (2009). Screening and Application of EST-Based PCR Markers Specific to Individual Chromosomes of *Haynaldia villosa*. Acta Agron. Sin..

[22] Liu C., Li S., Wang K., Ye X., Lin Z. (2017). Developing of Specific Transcription Sequences P21461 and P33259 on *Dasypyrum villosum* 6VS and Application of Molecular Markers in Identifying Wheat-*D. villosum* Breeding Materials with Powdery Mildew Resistance. Acta Agron. Sin..

[23] Bie T., Zhao R., Zhu S. (2015). Development and characterization of marker MBH1 simultaneously tagging genes *Pm21* and *PmV* conferring resistance to powdery mildew in wheat. Mol. Breed.

[24] Kreiner J.M., Kron P., Husband B.C. (2017). Evolutionary Dynamics of Unreduced Gametes. Trends Genet..

[25] Mason A.S., Pires J.C. (2015). Unreduced gametes: meiotic mishap or evolutionary mechanism?. Trends Genet..

[26] Liu C., Ye X., Wang M., Li S., Lin Z. (2017). Genetic behavior of *Triticum aestivum-Dasypyrum villosum* translocation chromosomes T6V#4S·6DL and T6V#2S·6AL carrying powdery mildew resistance. J. Integr. Agric..

[27] Zhang X., Chen S. (1990). Production and Utilization of Alien Substitution Lines of Common Wheat. Hereditas.

[28] Sears E.R. (1953). Nullisomic Analysis in Common Wheat. Am. Nat..

[29] Ghazali S., Mirzaghaderi G., Majdi M. (2015). Production of a novel Robertsonian translocation from *Thinopyrum bessarabicum* into bread wheat. Tsitol Genet.

[30] Danilova T.V., Friebe B., Gill B.S., Poland J., Jackson E. (2018). Chromosome Rearrangements Caused by Double Monosomy in Wheat-Barley Group-7 Substitution Lines. Cytogenet. Genome Res..

[31] Zhou S., Zhang J., Che Y., Liu W., Lu Y., Yang X., Li X., Jia J., Liu X., Li L. (2018). Construction of *Agropyron Gaertn*. genetic linkage maps using a wheat 660K SNP array reveals a homoeologous relationship with the wheat genome. Plant Biotechnol. J..

[32] Danilova T.V., Friebe B., Gill B.S. (2014). Development of a wheat single gene FISH map for analyzing homoeologous relationship and chromosomal rearrangements within the Triticeae. Theor. Appl. Genet..

[33] Zhang Y., Zhang J., Huang L., Gao A., Zhang J., Yang X., Liu W., Li X., Li L. (2015). A high-density genetic map for P genome of *Agropyron Gaertn*. based on specific-locus amplified fragment sequencing (SLAF-seq). Planta.

[34] Luo Z., Hackett C.A., Bradshaw J.E., McNicol J.W., Milbourne D. (2001). Construction of a genetic linkage map in tetraploid species using molecular markers. Genetics.

[35] Qi P., Eudy D., Schnable J.C., Schmutz J., Raymer P.L., Devos K.M. (2019). High Density Genetic Maps of Seashore Paspalum Using Genotyping-By-Sequencing and Their Relationship to The Sorghum Bicolor Genome. Sci. Rep..

[36] Jia J. (1996). Molecular Germplasm Diagnostics and Molecular Marker assisted Breeding. Scientia Agricultura Sinica.

[37] Qi L., Echalier B., Chao S., Lazo G.R., Butler G.E., Anderson O.D., Akhunov E.D., Dvorák J., Linkiewicz A.M., Ratnasiri A. (2004). A Chromosome Bin Map of 16,000 Expressed Sequence Tag Loci and Distribution of Genes Among the Three Genomes of Polyploid Wheat. Genetics.

[38] Zimin A.V., Puiu D., Hall R., Kingan S., Clavijo B.J., Salzberg S.L. (2017). The first near-complete assembly of the hexaploid bread wheat genome, *Triticum aestivum*. Gigascience.

[39] Lu F.H., McKenzie N., Kettleborough G., Heavens D., Clark M.D., Bevan M.W. (2018). Independent assessment and improvement of wheat genome sequence assemblies using Fosill jumping libraries. Gigascience.

[40] Mayer K.F., Waugh R., Brown J.W., Schulman A., Langridge P., Platzer M., Fincher G.B., Muehlbauer G.J., Sato K., International Barley Genome Sequencing Consortium (2012). A physical, genetic and functional sequence assembly of the barley genome. Nature.

[41] Ariyadasa R., Mascher M., Nussbaumer T., Schulte D., Frenkel Z., Poursarebani N., Zhou R., Steuernagel B., Gundlach H., Taudien S. (2014). A sequence-ready physical map of barley anchored genetically by two million single-nucleotide polymorphisms. Plant Physiol..

[42] Mascher M., Gundlach H., Himmelbach A., Beier S., Twardziok S.O., Wicker T., Radchuk V., Dockter C., Hedley P.E., Russell J. (2017). A chromosome conformation capture ordered sequence of the barley genome. Nature.

[43] Zhao R., Wang H., Xiao J., Bie T., Cheng S., Jia Q., Yuan C., Zhang R., Cao A., Chen P. (2013). Induction of 4VS chromosome recombinants using the CS ph1b mutant and mapping of the wheat yellow mosaic virus resistance gene from *Haynaldia villosa*. Theor. Appl. Genet..

[44] Li H., Deal K.R., Luo M., Ji W., Distelfeld A., Dvorak. J. (2017). Introgression of the *Aegilops speltoides* Su1-Ph1 Suppressor into Wheat. Front. Plant. Sci..

[45] Meng L., Li H., Zhang L., Wang J. (2015). QTL IciMapping: Integrated software for genetic linkage map construction and quantitative trait locus mapping in biparental populations. Crop J..

[46] Wei W.H., Qin R., Song Y.C., Ning S.B., Guo L.Q., Gu M.G. (2002). Location and analysis of introgressed segments in the parthenogenetic progenies of Zea mays×Z. diploperennis by GISH. Acta Bot. Sin..

